# Genetic effect of thyroid function-related diseases on acute respiratory distress syndrome: a Mendelian randomization study

**DOI:** 10.18632/aging.205996

**Published:** 2024-07-01

**Authors:** Peilong Li, Haifeng Liu, Feng Li, Mingze Sui, Kai Liu, Hongmin Fu

**Affiliations:** 1 Children’s Hospital Affiliated to Kunming Medical University, Kunming Medical University and Kunming Children’s Hospital, Kunming, P.R. China; 2 Department of Pulmonary and Critical Care Medicine, Kunming Children’s Hospital and Yunnan Key Laboratory of Children’s Major Disease Research, Kunming, P.R. China

**Keywords:** hypothyroidism, ARDS, Mendelian randomization, disorders of the thyroid gland, enrichment analysis

## Abstract

Background: Previous studies have shown an association between acute respiratory distress syndrome (ARDS) and thyroid function. However, their causal relationship remains unspecified. Therefore, this study aims to explore the causal relationship between ARDS and thyroid function-related diseases with Mendelian Randomization (MR) analysis.

Methods: ARDS dataset finn-b-J10_ARDS, finn-b-E4_THYROID dataset of disorders of the thyroid gland (DTG) and finn-b-E4_HYTHYNAS of hypothyroidism were acquired from public database. In univariate MR (UVMR), causal effects between DTG, hypothyroidism and ARDS were investigated using 5 types of algorithms, and reliability was validated by sensitivity analysis. Moreover, multivariate MR (MVMR), enrichment and interaction network analyses of genes corresponding to SNPs of DTG and hypothyroidism were carried out. Significant level was chosen as p<0.05.

Results: UVMR identified DTG and hypothyroidism (*P* < 0.05, *OR* > 1) as risk factors, and were causally related to ARDS. Reliability of UVMR results was confirmed through sensitivity analysis, and results were stable and reliable. However, DTG and hypothyroidism had no effect on ARDS in MVMR, possibly because these factors had independent effects on ARDS. Ultimately, 96 and 113 genes corresponding to SNPs of DTG and hypothyroidism were found closely related to immune-related pathways.

Conclusions: UVMR and MVMR analysis revealed a causal connection between DTG and hypothyroidism as risk factors with ARDS, providing robust evidence for investigation into relationship of hypothyroidism on ARDS and between DTG and ARDS.

## INTRODUCTION

Acute respiratory distress syndrome (ARDS) manifests as hypoxemic respiratory failure and bilateral lung infiltrates due to reduced alveolar fluid clearance (AFC), often requiring mechanical ventilation [1]. Edema seen in ARDS results from epithelial and endothelial damage, leading to increased epithelial permeability and diminished AFC [2]. Coronavirus disease 2019 (COVID-19), caused by severe acute respiratory syndrome coronavirus 2 (SARS-CoV-2), has rapidly spread globally, posing a significant health risk [3]. During pandemic, around 5% of severe COVID-19 individuals necessitated intensive care unit (ICU) admission, with two-thirds progressing to ARDS [4]. Despite extensive research, the mortality rate remains higher, ranging from 35 to 46 percent [5]. Currently, there are no approved specific molecular therapies for ARDS, and standard of care involves mechanical ventilation, supplemental oxygen, and supportive measures [6].

Recent research suggests that thyroid hormones can decrease fibrin deposition in murine models of pulmonary fibrosis induced by both bleomycin and inducible TGF-β1 [7]. Currently, a preclinical safety study is underway to explore potential of administering thyroid hormones directly into the lungs of healthy rats as a therapeutic strategy for ARDS. ARDS is recognized as a condition that can lead to multi-organ injury, with lungs being primary target organ. Consequently, there is a presumption that ARDS may have adverse effects on thyroid gland. However, the available literature on clinical observations, particularly data obtained from blood samples of ARDS patients examined for thyroid function, is limited. Adenohypophyseal endocrine cell destruction is presumed in ARDS patients. Hypothyroidism is characterized by inadequate production of thyroid hormones, which can be congenital and arise from mutations in proteins essential for the synthesis pathway of thyroid hormones, defects in the formation of the thyroid gland, or even the absence of the gland [8].

Currently, the precise causal connection between hypothyroidism and ARDS remains a subject of ongoing debate within the academic community. Furthermore, the methodological constraints inherent in observational research, including the possibility of biases and confounding variables, persist as a significant area of apprehension. Therefore, it is imperative to examine the causal connection by employing an appropriate approach and a substantial sample size.

Mendelian randomization (MR) is a method that utilizes genetic variation as a natural experimental tool to study impact of environmental factors on outcomes, like disease risk [9]. Alleles assort randomly during meiosis, independent of disease risk and unaffected by ascertainment bias. MR leverages this to ascertain whether the risk of a particular disease depends on genetic predisposition to exposure to a risk factor. Given random transmission of genotypes from parents to offspring, the relationship between genetic variants and outcomes remains unswayed by common confounders, rendering causal inference plausible [10]. MR offers a valuable approach for analyzing causal relationships in ARDS, overcoming challenges common in observational research, such as selection bias and limitations due to small sample sizes. This method is not constrained by sample size, as exposure is typically measured in an independent cohort regarding the outcome (ARDS), given that cohorts demonstrate comparable genetic structures, often population-matched. Hence, there’s no need to obtain two separate data points (exposure and outcome) for each patient, which is a common bottleneck in observational studies. Utilization of independent sample sets is referred to as UVMR [11]. Commonly used approach to conduct MR is utilizing summary statistics from public dataset. The accessibility of statistics has significantly increased due to emergence of large biobanks that incorporate publicly available data [12]. In an exposure GWAS, SNPs associated with proposed risk factor are identified. These SNPs serve as instruments to measure genetic liability to exposure of interest. Subsequently, instrumental SNPs are assessed in a GWAS to ascertain relationship between exposure and disease risk. A causal inference is drawn based on a linear relationship observed between exposure, measured by the instrument, and the risk of disease outcome.

In this study, we employed UVMR and MVMR approach to investigate potential genetic and causal relationship between hypothyroidism and DTG with ARDS [13].

## MATERIALS AND METHODS

### Data sources

### Overall data declaration

Finn-b-J10_ARDS dataset of ARDS as outcome, Finn-b-E4_THYROID dataset of disorders of the thyroid gland (DTG) and Finn-b-E4_HYTHYNAS of hypothyroidism as exposure factors were obtained from Open GWAS database (https://gwas.mrcieu.ac.uk/). Finn-b-J10_ARDS dataset included 16,380,461 SNPs data, Finn-b-E4_THYROID dataset included 16,380,466 SNPs and Finn-b-E4_HYTHYNAS included 16,380,461 SNPs.

### Selection of associated SNPs

To estimate causal effects, genetic variants were extracted as instrumental variables (IVs) based on following hypotheses: (1) predictive of hypothyroidism and DTG, (2) unaffected by confounding factors, and (3) not influenced ARDS through another pathway except hypothyroidism and DTG. It is quite important that these principles are firstly met.

### Screening of IVs

To fulfil the first hypothesis, hypothyroidism and DTG-associated SNPs were selected at p < 5 × 10^−8^ from corresponding dataset. Then, linkage disequilibrium (LD) was executed to ensure SNPs were independent (*r^2^* = 0.001, distance = 10000 kb). After screening, 43 SNPs of DTG and 51 SNPs of hypothyroidism were obtained. Next, PhenoScanner database (http://www.phenoscanner.medschl.cam.ac.uk/) was further used to verify presence of confounders in selected SNPs. Later, *F* statistics for SNPs was calculated to exclude weak IVs (*F* = (*N*-*κ*-1)*R^2^/ [κ*(1-*R^2^*)], *N*: sample size, *κ*: the number of IVs, and *R^2^*: proportion of variation in hypothyroidism and DTG for each SNP; *R^2^* = *β*^2*2MAF* (1-*MAF*), *β*: the effect size of SNPs, *MAF* (minor allele frequency). Finally, SNPs with *F* > 10, *MAF* < 0.3 were selected for subsequent analyses. Of these, 40 SNPs of DTG and 50 SNPs of hypothyroidism were used for MR analysis.

### MR statistical analysis

Harmonise effect equivalents and sizes were performed by harmonise data in TwoSampleMR package. MR analysis was conducted with MR-Egger [14], Weighted median [15], Inverse variance weighted (IVW) [16], Simple mode [17] and Weighted mode [18], and the primary analysis was performed using IVW. Odds Ratio (OR) was then calculated. Exposure factors with *OR* > 1 were risk factors and *OR* < 1 were protective factors. MVMR analysis was conducted to explore combined effect of hypothyroidism and DTG on ARDS.

### Sensitivity analysis

### Heterogeneity analysis

Cochran’s *Q* test was employed to evaluate presence of heterogeneity among SNPs using MR Egger and IVW methods. If *P* value > 0.05, indicating absence of heterogeneity, fixed-effects IVW method was chosen as primary method. Otherwise, random-effects model was utilized.

### Horizontal pleiotropy analysis

One assumption of MR was that IVs must influence outcome solely through exposure factor. If IVs could directly affect results without involving exposure factor, it suggests horizontal pleiotropy, violating MR assumption. Therefore, we used ‘mr_pleiotropy_test’ function to test for the presence of horizontal pleiotropy. *P* value > 0.05 indicated absence of horizontal pleiotropy, ensuring reliability of results.

### Leave-one-out (LOO) sensitivity test

LOO test was performed by sequentially removing each SNP and assessing effect of remaining SNPs on outcome. If results changed significantly after removing a specific SNP, it suggested sensitivity of the outcome to that SNP. We calculated meta-effect of remaining SNPs after eliminating of each SNP using ‘mr_leaveoneout’ function.

### Functional analysis of genes associated with SNPs

Firstly, we retrieved key genes 1 and 2 corresponding to SNPs associated with DTG and hypothyroidism using eQTLGen database. Then, enrichment analysis of key genes 1 and 2 was performed using ‘clusterProfiler’ package [19] (*P*.adj<0.05). At last, interactions of key genes 1 and 2 in protein level were comprehended through GeneMAINA database.

**Figure 1:**
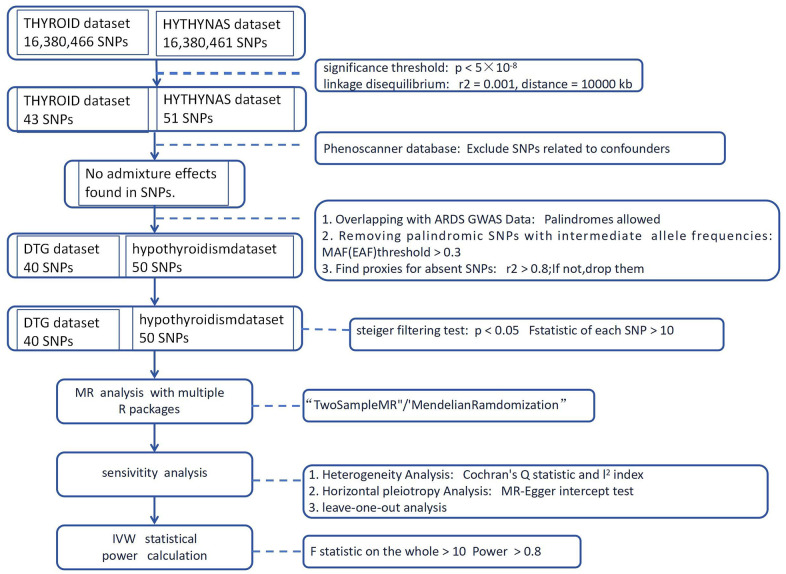
Flow diagram for whole MR analysis.

MR analyses were performed using *R* software (version 4.2.3), primarily ‘TwoSampleMR’ (version 0.5.6). Additional R packages were employed for analysis conducted by helper. Entire procedure was illustrated in Figure 1.

### Availability of data and materials

All the data obtained and materials analyzed in this research are available from the corresponding author upon reasonable request.

### Consent for publication

All authors have read and agreed to the published version of the manuscript.

## RESULTS

### DTG and hypothyroidism as risk factors influenced development of ARDS

Weight median is upgraded of MR-Egger, designed to statistical deficiencies present in MR-Egger method [20]. Marked distinction of IVW and MR-Egger regression lies in whether “intercept” is in respective calculation formulas, which often leads to disparate results. Typically, inconsistencies of two results arise due to limitations in statistical methods and lower power of MR-Egger [21]. After filtrating, 40 SNPs of DTG and 50 SNPs were acquired as IVs for UVMR analysis, respectively (Supplementary Tables 1, 2). UVMR results from IVW indicated ARDS had causality with DTG and hypothyroidism (*P* < 0.05), DTG (*OR* = 1.672) and hypothyroidism (*OR* = 1.465) were identified as risk factor (Table 1). Scatter plots appeared positive correlation of DTG and hypothyroidism with ARDS, again supporting these results (Figure 2A). The forest plot illustrated that DTG and hypothyroidism similarly increased the risk of morbidity (Figure 2B). Valid SNPs in UVMR were symmetrically distributed in funnel plots, which aligning with Mendel’s second law (Figure 2C).

**Table 1:** Causal assessment in each MR method.

**MR Method**	**Disorders of the thyroid gland**					**Hypothyroidism**				
	***β***	***SE***	***p***	***OR***	***95%CI***	***β***	***SE***	***p***	***OR***	***95%CI***
MR-Egger	0.34	0.45	0.45	1.40	(0.58,3.41)	0.66	0.40	0.11	1.93	(0.88,4.23)
Inverse variance weighted	0.51	0.19	0.01	1.67	(1.13,2.46)	0.38	0.17	0.02	1.46	(1.06,2.02)
Weighted median	0.39	0.28	0.16	1.49	(0.86,2.58)	0.35	0.25	0.16	1.42	(0.87,2.31)

**Figure 2:**
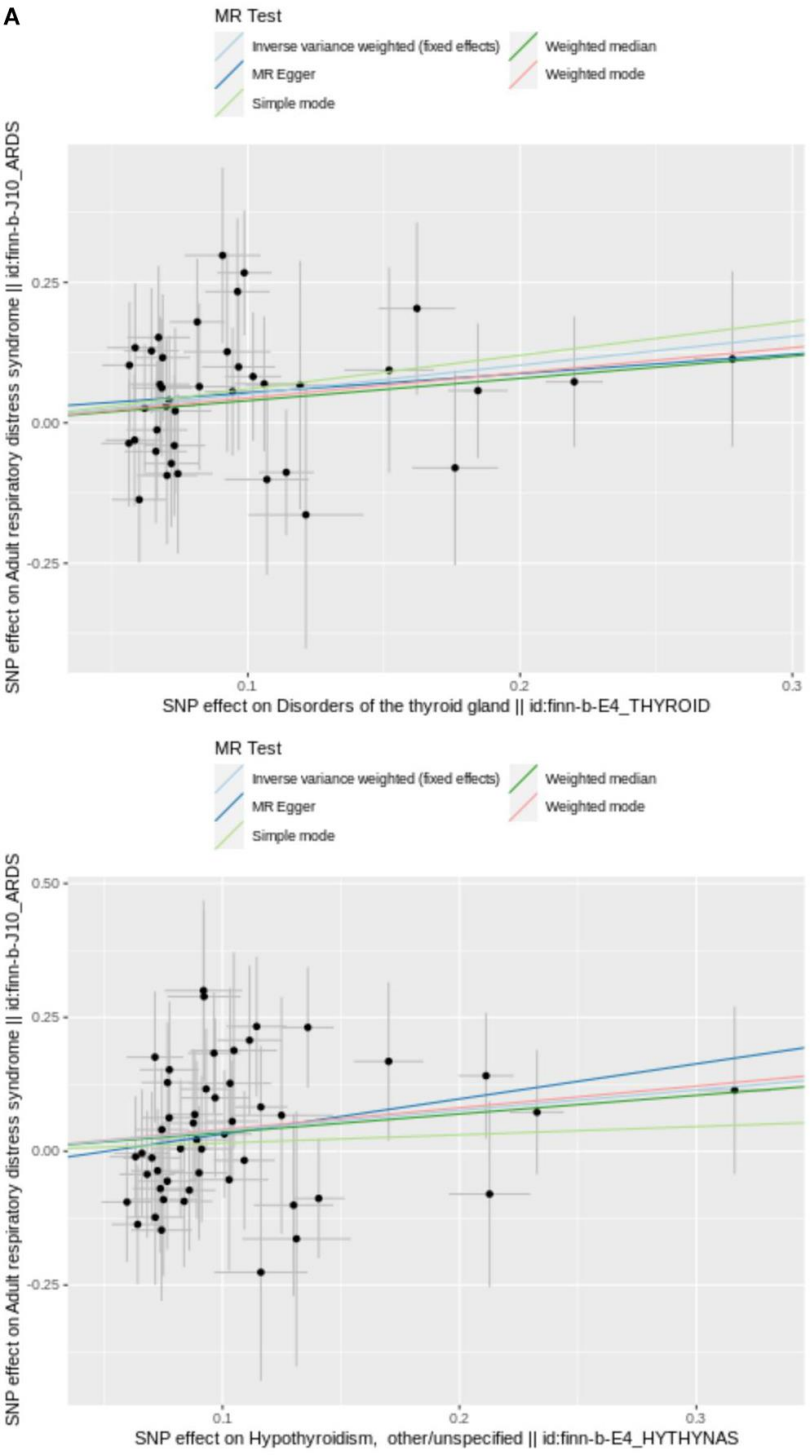
(**A**) Scatter plot of SNPs relevant to DTG, hypothyroidism and ARDS. Each splash displayed the effect sizes for SNP relation (x-axis, SD units) and SNP-ARDS relation (y-axis, log (OR)) with 95% CIs. Using three MR strategies (IVE, MR-Egger, and weighted median), the regression slopes of the lines associated with the causal estimates were determined.

**Figure 2:**
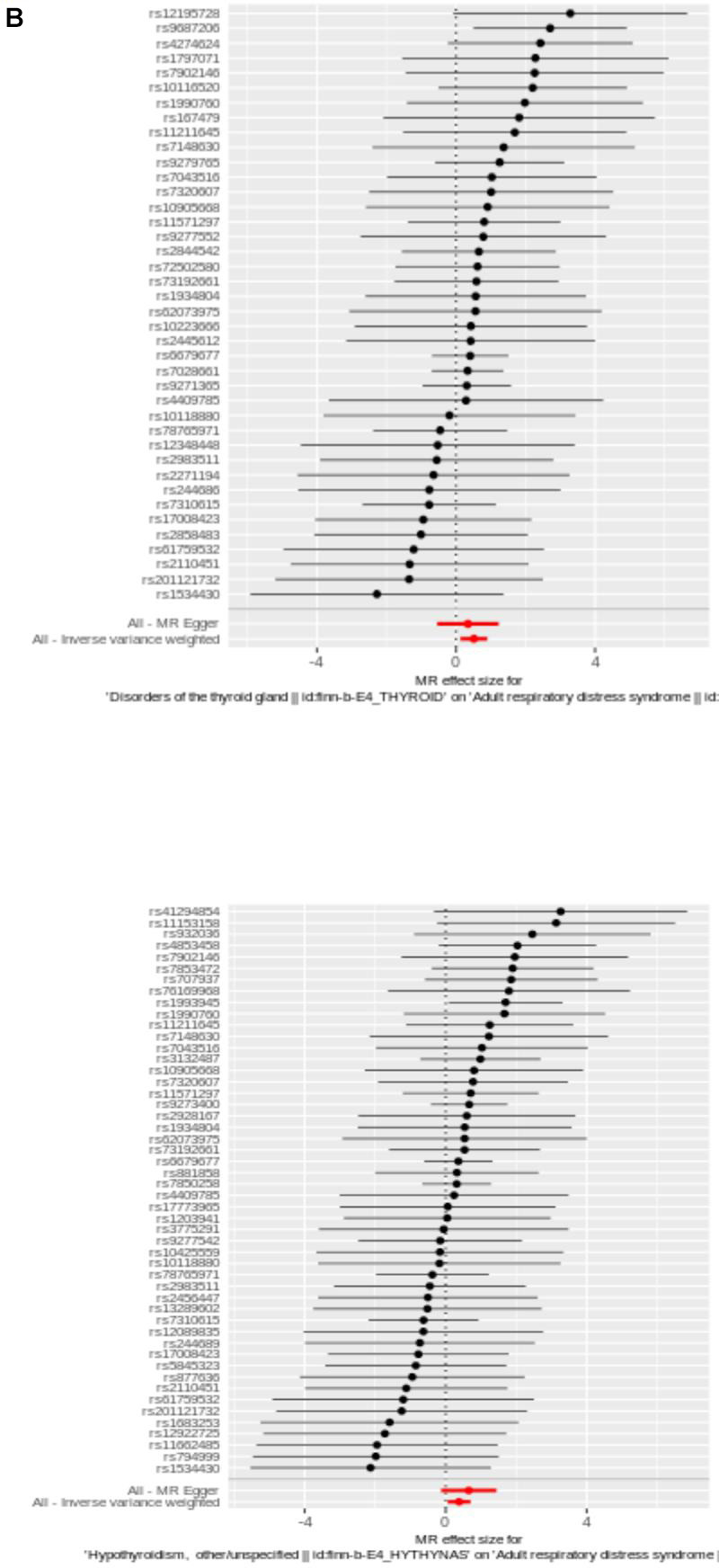
(**B**) The utilization of a funnel plot. The utilization of a funnel plot as a means to assess the robustness of a study is being considered. The dispersed data points represent the approximate impact of a solitary SNP employed as an IV. Global estimate was depicted by the vertical lines, which were obtained using IVW approach using MR-Egger regression.

**Figure 2:**
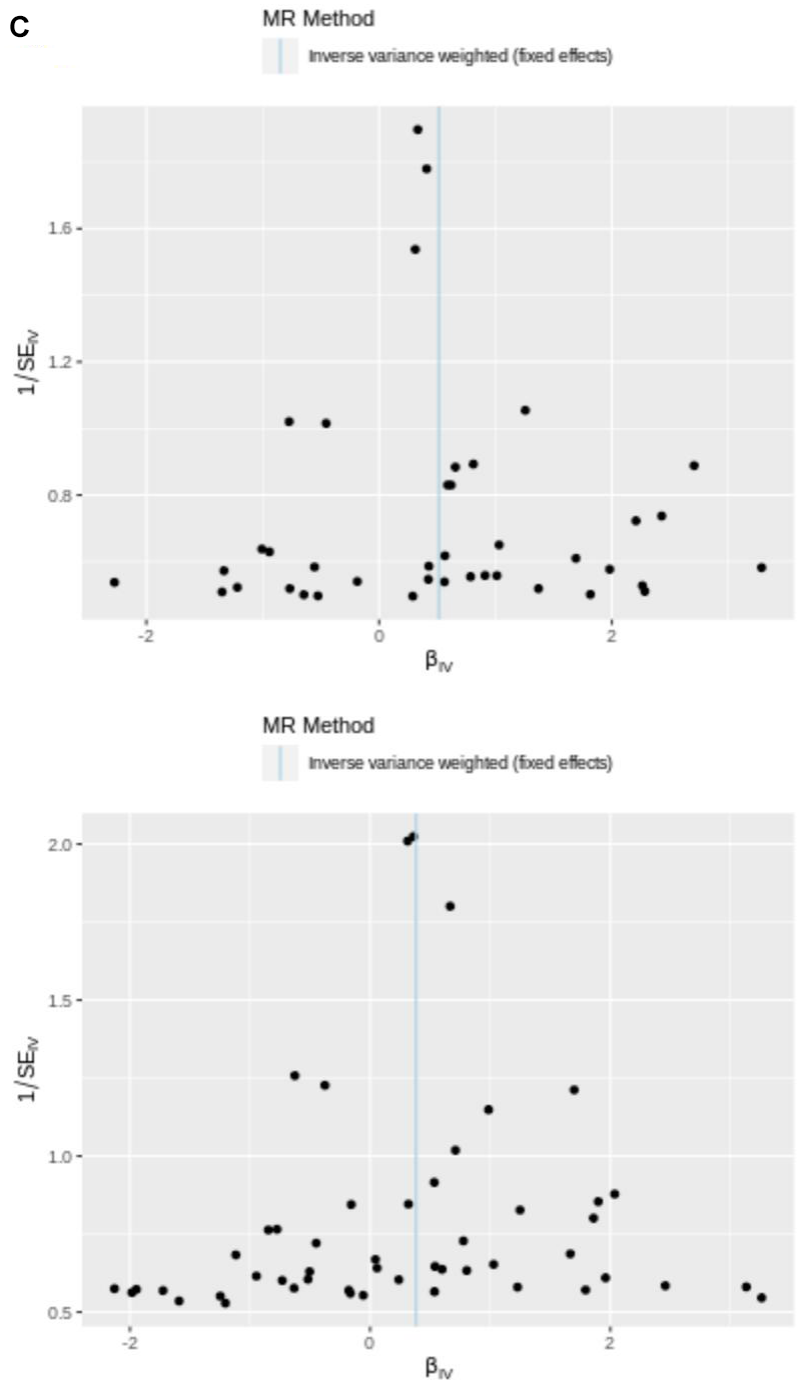
(**C**) IVW line in funnel plot. SNPs exhibited a symmetrical distribution on either side of IVW line in funnel plot. This observation suggests that MR analysis adhered to Mendel’s second law.

**Figure 2:**
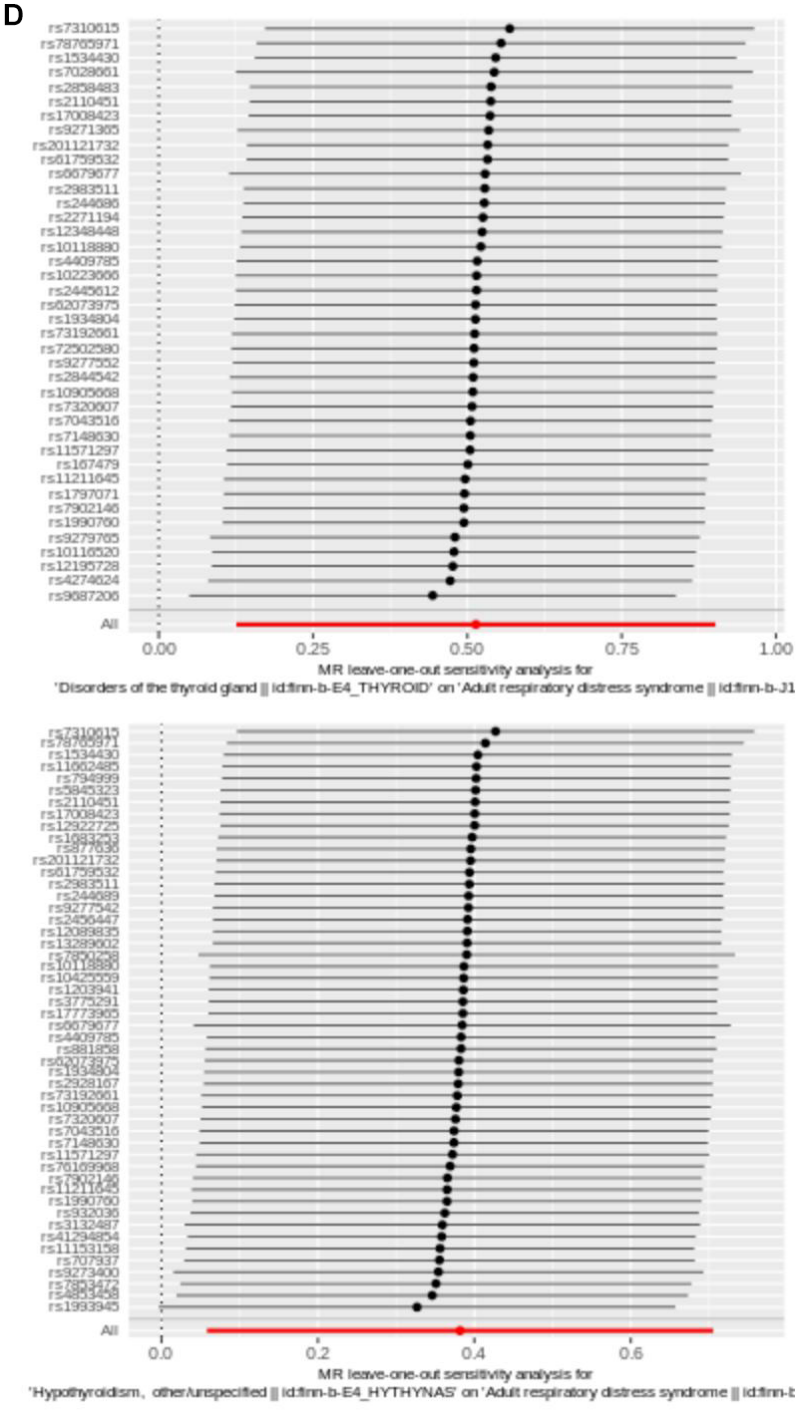
(**D**) Plot of LOO. Each row was considered an independent MR analysis to estimate causal effect using all of remaining IVs except for single SNP listed on y-axis. Additionally, all data points were required to be greater than zero on the x-axis.

Next, reliability of UVMR results was evaluated by sensitivity analysis. *Q* value > 0.05 for the test of heterogeneity indicated that there was no heterogeneity between the samples. Then, results displayed no horizontal pleiotropy (*P* > 0.05) (Table 2). In addition, absence of significant bias points of LOO analysis suggests that findings were reliable (Figure 2D).

**Table 2:** Heterogeneity and pleiotropy tests of IVs.

	**Cochran’s *Q* test**			
	**MR-Egger**		**Inverse variance weighted**	
	***Q***	***p***	***Q***	***p***
**DTG**	25.64	0.94	25.81	0.95
**Hypothyroidism**	35.91	0.90	36.47	0.91

### DTG and hypothyroidism had no effect on ARDS

After screening, 79 independent SNPs related to DTG and hypothyroidism were screened out as IVs for MVMR analysis. The MVMR results showed that DTG and hypothyroidism had no effect on ARDS, possibly because the two factors had independent effects on ARDS (Table 3).

**Table 3:** MVMR analysis.

**MVMR**	**ARDS**				
	**SNP**	***β***	***p***	***OR***	***95%CI***
**Hypothyroidism**	46	-0.36	0.79	0.69	(0.04,9.72)
**DTG**	33	0.96	0.55	2.60	(0.12,58.19)

*Abbreviations: MVMR, Multivariable MR analysis.

### Sensitivity analysis

### Heterogeneity analysis

We calculated Cochran’s Q statistic to detect IVs with potential heterogeneity as shown in Table 2. We found that no heterogeneity among final 40 SNPs of DTG and 50 SNPs (IVW: *p* > 0.05).

### Horizontal pleiotropy analysis

To lower the bias produced by horizontal pleiotropy, MR-Egger intercept test following a linear model was further performed (hypothyroidism: *p* = 0.108 > 0.05, DTG: *p* = 0.0.454 > 0.05). Visual results of all four methods were displayed in a scatter plot, with the intercept of MR-Egger regression model shown (Figure 2A). The Figures showed the essentially symmetrical distribution in the two main MR approaches of increased genetic effect estimates of DTG and hypothyroidism on ARDS risk when a single SNP was employed as IV. However, statistical power of this study was relatively insufficient, which might be caused by deficiency of the case size.

### Genetic effects of a single IV for ARDS

LOO was undertaken to confirm impact of each SNP on overall causal estimate. When methodically removing SNPs and rerunning MR analysis, there was no discernible change in the predicted causal effect here (Figure 2D). Therefore, none of individual genetic instrument among these SNPs had a substantial influence on final MR outcomes.

### Genes corresponding to obtained SNPs were related to immunity

Enrichment analysis was conducted by 96 key genes 1 and 113 key genes 2 to find corresponding functions and pathways (Supplementary Tables 3, 4), these genes were incorporated into PPI network (Figure 3A, 3B). Enrichment results of key genes 1 showed that a large number of T cell activation regulation, proliferation regulation and other related functions were enriched (Figure 3C). They were also enriched in metabolism-related pathways, such as arginine and proline metabolism (Figure 3D). GO results showed that key genes 2 were predominantly enriched in immune-related pathways, particularly those immune response regulation, immune response regulation, monocyte differentiation, etc. (Figure 3E). And KEGG results showed those genes were enriched in T-cell receptor signalling pathway, which was crucial for immunity (Figure 3F).

**Figure 3:**
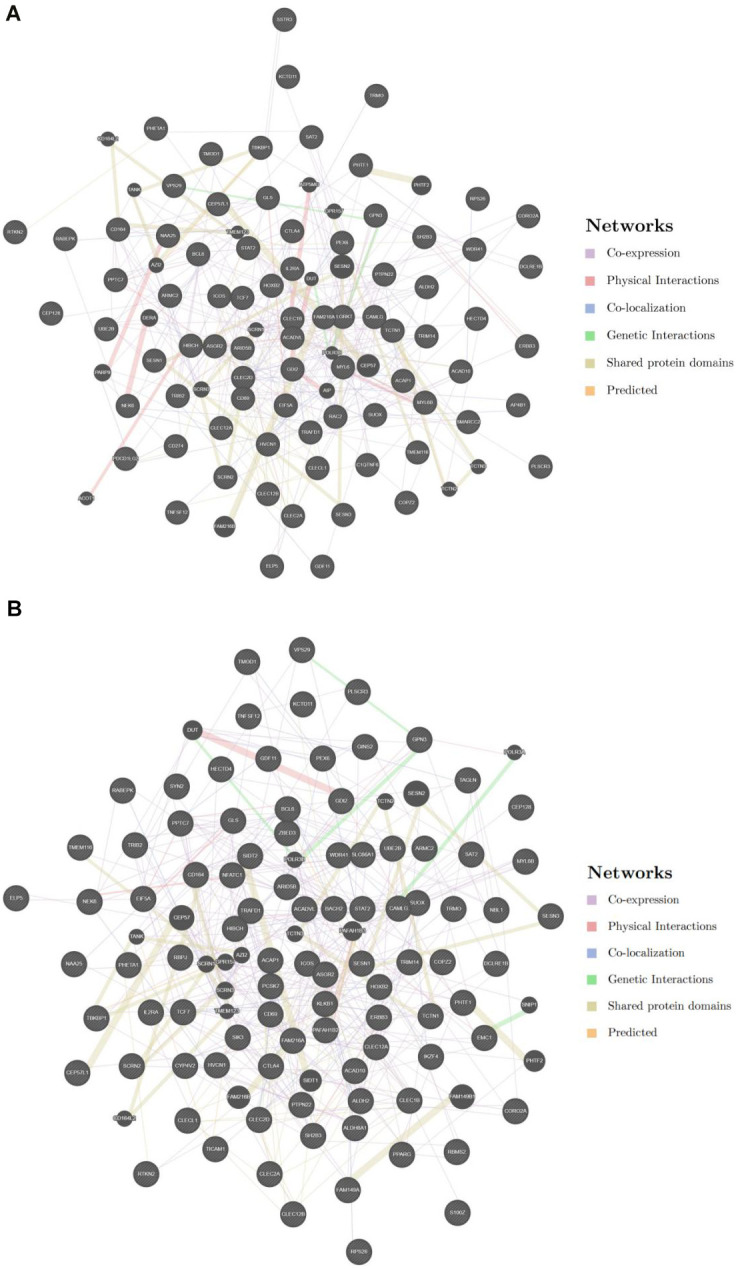
(**A**) PPI network among key genes 1. (**B**) PPI network among key genes 2.

**Figure 3:**
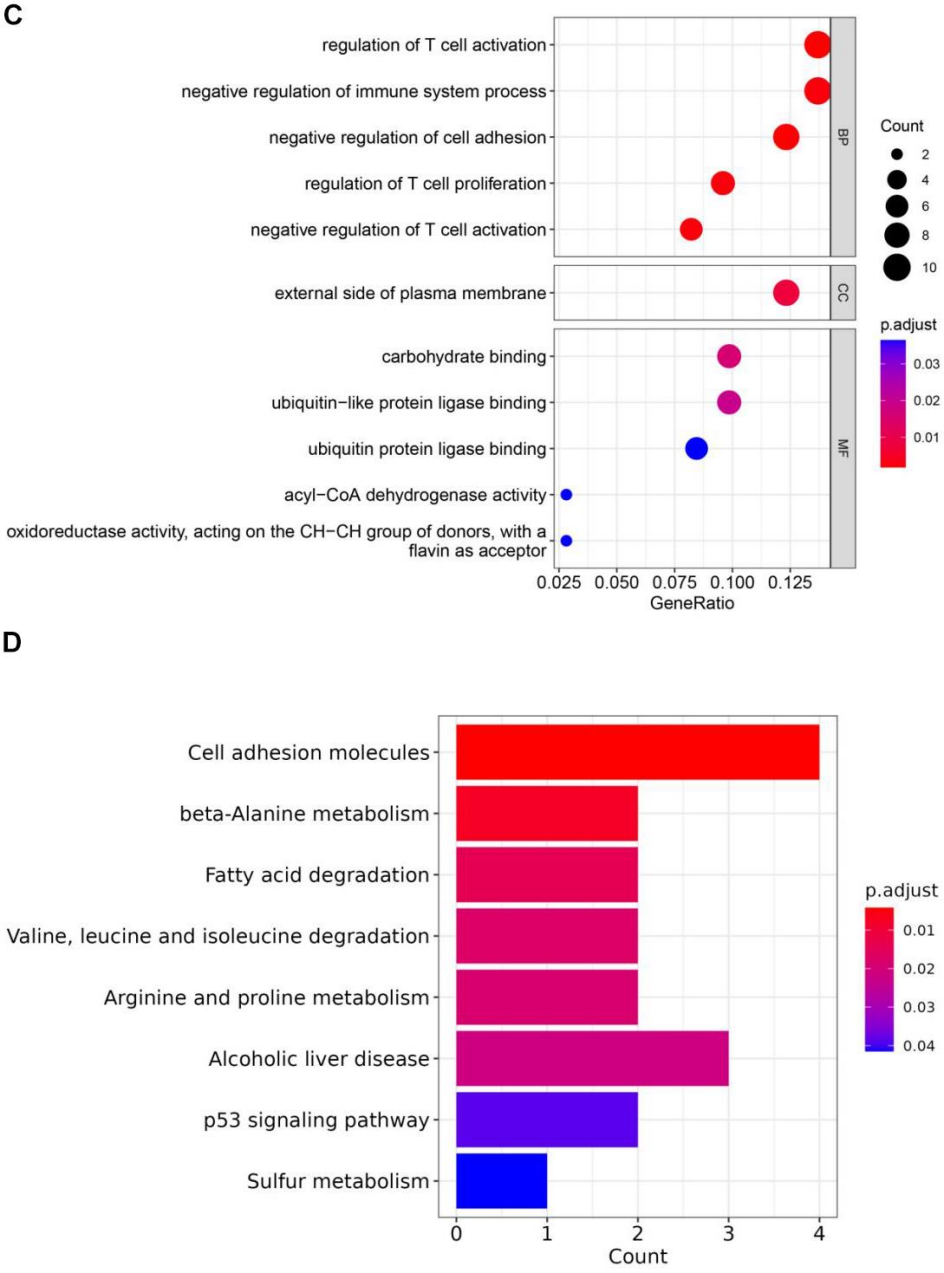
(**C**) GO enrichment analysis of key genes 1. (**D**) KEGG enrichment analysis of key genes 1.

**Figure 3:**
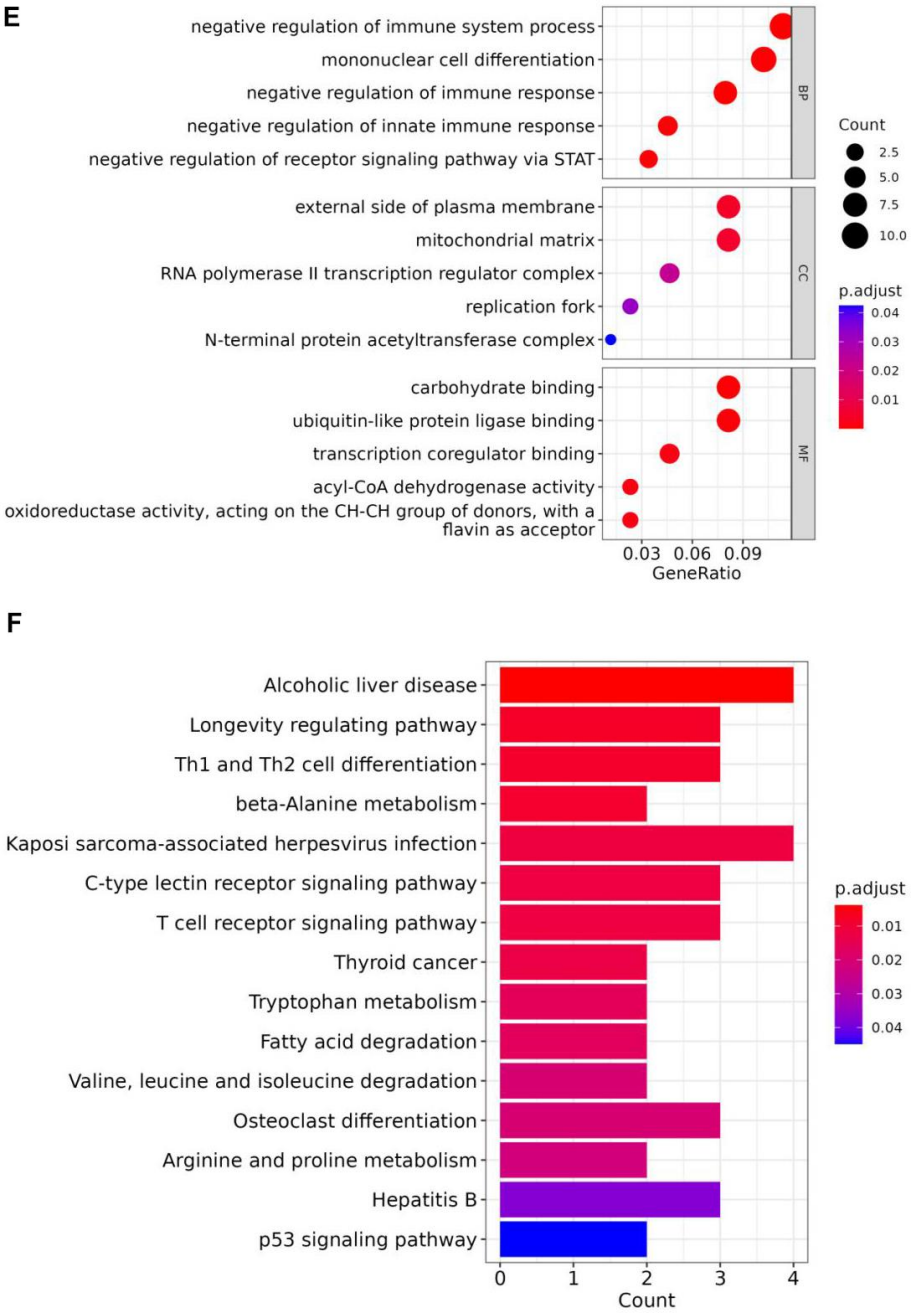
(**E**) GO enrichment analysis of key genes 2. (**F**) KEGG enrichment analysis of key genes 2.

## DISCUSSION

In this study, we utilized GWAS datasets and employed MR analysis to investigate the influence of genetic variation in DTG and hypothyroidism on the susceptibility to ARDS. We revealed the relationship using UVMR analysis and perform MVMR analysis. Probable evidence has been discovered, establishing a potential association between genetic variation DTG, hypothyroidism and an elevated susceptibility to ARDS.

ARDS is a severe respiratory system disorder, and Acute Physiology and Chronic Health Evaluation II (APACHE II) score is commonly employed to assess severity of acute illness, including patients with ARDS. On the other hand, Sequential Organ Failure Assessment (SOFA) score is utilized to monitor changes in organ function in ICU, especially in patients exhibiting evident clinical symptoms such as ARDS. Elevated SOFA scores typically indicate severe organ dysfunction. These scoring tools are frequently utilized in the ICU to evaluate the severity of ARDS and the response to treatment [22]. Research indicates that the observation of serum fT3 functions as a robust predictor mortality in ARDS patients [23]. Other studies have also suggested that the combined assessment of serum fT3 levels and APACHE-II score provides most reliable means for predicting patients’ mortality in ICU with ARDS [24]. In a study involving 206 patients with ARDS, prognostic efficacy of serum fT3, fT4, and TSH was explored [25].

Similarly, some patients with thyroid diseases, the occurrence of DTG is relatively common [26]. The severity of DTG may be influenced by many factors (tissue, nature, and severity of thyroid diseases). These influences encompass changes in thyroid hormone binding protein levels and binding affinity, alterations in thyroid hormone transporter dynamics, adjustments in expression and activity of thyroid hormone deiodinases, as well as variations in the expression of peripheral thyroid hormone receptors [27]. ARDS patients which have complication of DTG, the resulting outcomes have not been thoroughly investigated. Rothberger GD et al. studies convened 162 patients with ARDS who also had the complication of DTG and underwent mechanical ventilation [28]. In the study, serum fT3 levels were measured on day of initiation of mechanical ventilation. Study evaluated mortality rate and the number of ventilator-free days (VFDs) on day 28 after initiation of mechanical ventilation. Patients with low level (<2.3 pg/mL) exhibited a significantly higher mortality rate (52% vs. 19%), and significantly fewer mean and median VFDs on day 28 (10.7 vs. 18, and 0 vs. 23) compared with normal group. The study illustrated thyroid hormone level abnormalities caused by DTG are associated with adverse outcomes in ARDS patients requiring mechanical ventilation.

Our study performed UVMR and MVMR analyses. In UVMR analysis, we investigated causal relationships of hyperthyroidism and DTG on ARDS. Stability and reliability of these results confirmed through sensitivity analysis, and it was discovered that some SNPs were strongly associated with hypothyroidism. Consequently, MVMR analysis was undertaken to consider both DTG and hypothyroidism as exposures. Unfortunately, MVMR result did not prove findings of UVMR analysis, suggesting that DTG and hypothyroidism may indeed have an effect on ARDS. Anyway, our study revealed DTG and hypothyroidism are two causes of ARDS. Based on the findings from the above studies, we formulate a statement that the onset and persistent occurrence of ARDS are closely associated with the affected thyroid induced by DTG and hypothyroidism. Significance of our study lies in alerting physicians and patients to monitor thyroid function closely following an ARDS diagnosis. Early detection and treatment of thyroid disorders could help reduce overall bodily harm ARDS is a severe lung complication that poses a significant risk to life, arising from a state of systemic inflammation. Above findings indicate that there is a need to assess the potential association between hypothyroidism and the occurrence of ARDS.

Our investigation had several merits. First, our study was firstly researched causality of ARDS with hypothyroidism and DTG using UVMR and MVMR. Furthermore, strength of IV enabled us to conduct foremost MR analysis with positive outcome. Given the limited availability of relevant MR studies, this represents a pioneering and immensely impactive endeavor aimed at investigating whether genetic predisposition to DTG and hypothyroidism might elevate the risk of developing ARDS. This question was neither independently nor exhaustively investigated. IVs in our study were independent of confounding factors in relationship, thereby avoiding violations possibility of fundamental and strict MR assumptions. In the interim, implementation of MR methods would significantly mitigate impact of reverse causality compared with observational studies, to considerable extent. Furthermore, our study employed valid IVs represented by SNPs with robust associations and substantial intensity. This approach ensured a high degree of comparability between exposure and outcome samples, bolstering the credibility of our conclusions. Notably, building on insights gained from UVMR analysis, we conducted a MVMR analysis, adopting a cautious approach and refraining from uncritically accepting the results of UVMR analysis.

Our study also lacked some flexibility: (1) data in this paper are all from FinnGen database, which may run the risk of overlapping samples. Due to unavailability of individual-level information in secondary data, we were unable to adjust for gender and survival biases. So, we removed SNPs linked with survival proxied by age at enrollment to mitigate this bias; (2) MR approach operates under the assumption of a linear correlation between exposure and outcome, thus it cannot assess the non-linear relationship between hypothyroidism and the risk of ARDS. Moreover, moderate result from MR-Egger regression may suggest presence of unidentified confounding factors in exposure-outcome link; (3) applicability of our study was intended to extend to other ancestries; (4) power of this study may have been inadequate, likely due to an insufficient sample size, particularly in exposure cases. Moreover, after revealing no causal relationship between hypothyroidism with ARDS and DTG with ARDS through MVMR, we refrained from conducting additional analyses on other potential exposures in the context of ARDS.

## CONCLUSIONS

Study suggests that DTG and hypothyroidism was closely associated with the onset of ARDS. On basis of these findings, we recommended that individuals with DTG and hypothyroidism should undergo early treatment to reduce the ARDS risk. Fortunately, DTG and hypothyroidism could be diagnosed with some specific clinical diagnostic criteria and are treatable under most circumstances. More importantly, this study partly inspires the future studies on thyroid-ARDS relationship. These findings could potentially aid in furthering mechanistic and therapeutic studies concerning the relationship between thyroid function and ARDS, as well as understanding their systemic chain of biological activities.

## Supplementary Material

Supplementary Table 1

Supplementary Table 2

Supplementary Table 3

Supplementary Table 4
